# P-1025. A Systematic Literature Review and Bayesian Meta-analysis of Clostridioides difficile Primary Prophylaxis with Vancomycin in Stem Cell Transplant Patients

**DOI:** 10.1093/ofid/ofaf695.1221

**Published:** 2026-01-11

**Authors:** Wajeeha Tariq, Erika P Viana-Cardenas, Eugenia Miranti, Sa Shen, Guillermo Rodriguez-Nava, Mingjun Jiang, Mindy M Sampson, Jorge Salinas, Alexandre Marra

**Affiliations:** Stanford University, Palo Alto, CA; Stanford University, Palo Alto, CA; Stanford Medicine, Stanford, CA; Quantitative Sciences Unit, Stanford, California; Stanford University School of Medicine, Stanford, California; Stanford University, Palo Alto, CA; Stanford University, Palo Alto, CA; Stanford University, Palo Alto, CA; University of Iowa Hospital and Clinics, iowa city, Iowa

## Abstract

**Background:**

*Clostridioides difficile* infection (CDI) is common among patients undergoing hematopoietic stem cell transplantation (HSCT). Oral vancomycin prophylaxis may effectively prevent CDI in certain populations. We investigated the effectiveness of primary oral vancomycin prophylaxis in preventing CDI in HSCT patients.

**Figure 1.**
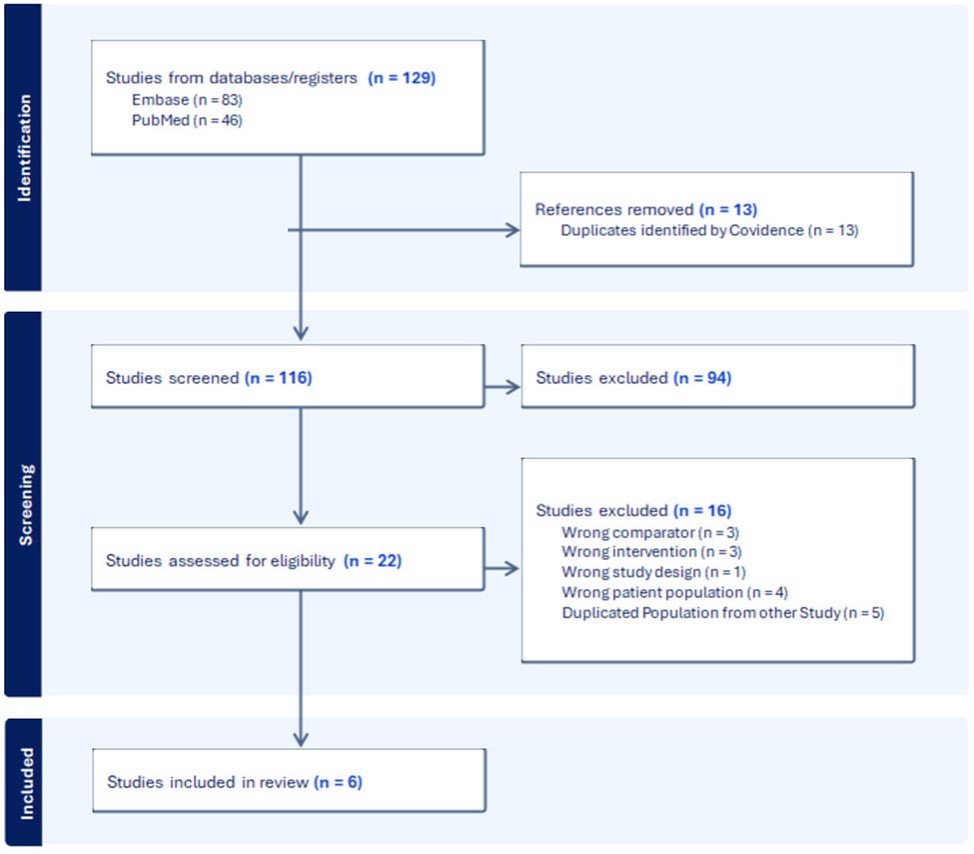
Literature search for studies that evaluated the impact of Oral Vancomycin Prophylaxis for Clostridioides difficile Infection in Transplanted Population.

**Figure 2.**
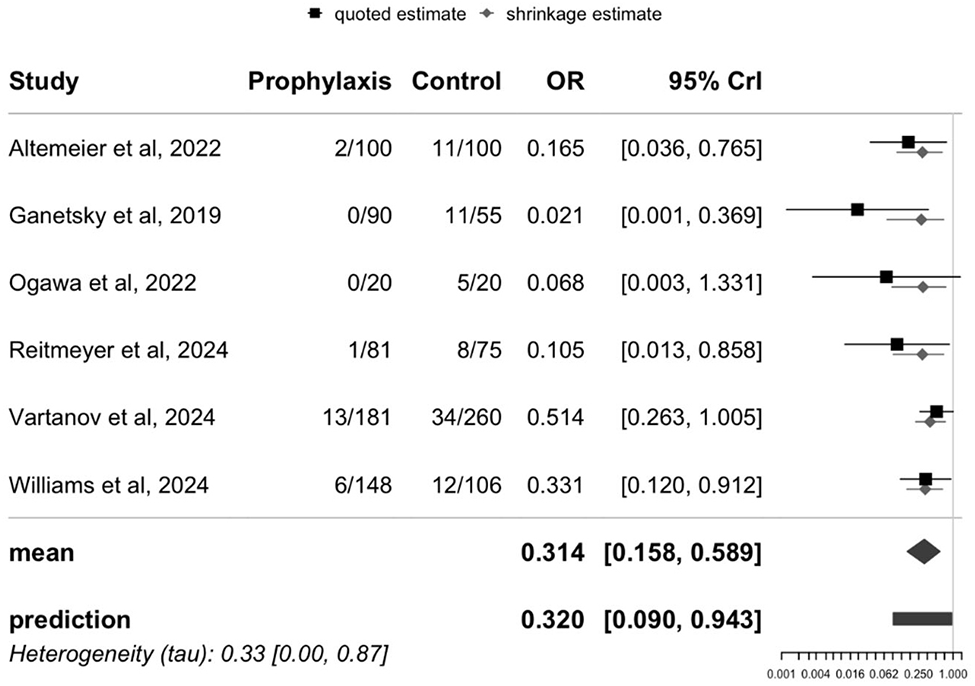
Forest plot of Clostridioides difficile Infection with and without Oral Vancomycin Prophylaxis.

**Methods:**

We conducted a Bayesian meta-analysis. PubMed and Embase databases were searched from inception to March 21, 2025. We included studies comparing the incidence of CDI in HSCT recipients who received primary oral vancomycin prophylaxis versus those who did not. We used the Downs and Black scale for quality assessment. Publication bias was assessed using Robust Bayesian Meta-Analyses and heterogeneity was also calculated. Effect sizes and standard errors were calculated from the sample sizes and reported cases, and a Bayesian random-effects model was used to conduct the meta-analysis. The primary outcome was the frequency of CDI in patients who received primary oral vancomycin prophylaxis compared to those who did not. Secondary outcomes included incidence of positive VRE cultures (from any site), blood stream infections, GVHD, and length of hospital stay. The review was registered on PROSPERO (CRD420251016925).

**Figure 3.**
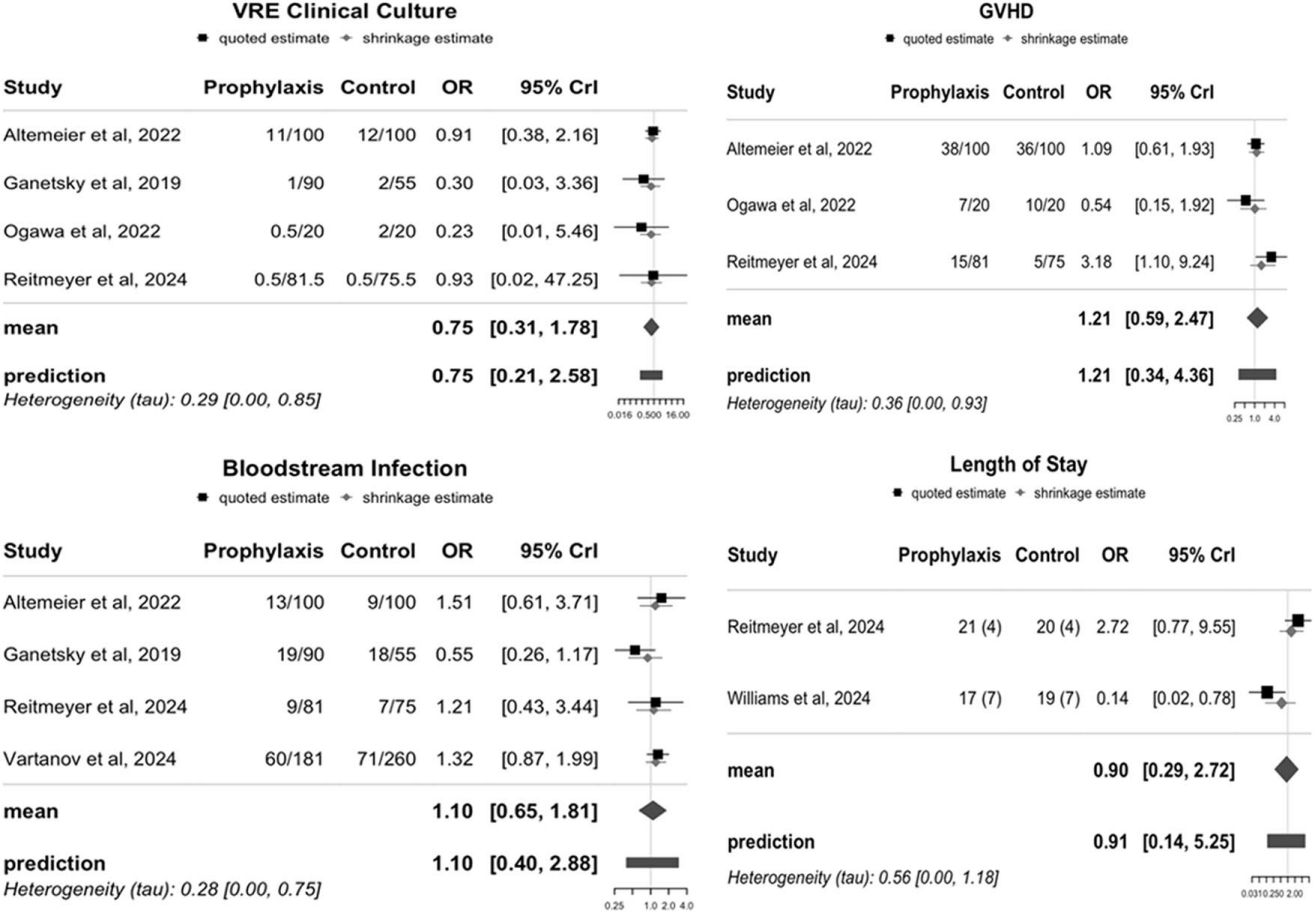
Forest plot of Secondary Outcomes with and without Oral Vancomycin Prophylaxis.

**Results:**

Six studies (including 2 abstracts) met inclusion criteria with a total of 1,236 patients (620 in the intervention group and 616 in the control). The 4 full-text studies were high quality. Primary oral vancomycin prophylaxis reduced the incidence of CDI (OR 0.31; CrI 0.16–0.59). Secondary outcomes including bloodstream infections (OR 1.10; CrI 0.65–1.81), positive VRE cultures (OR 0.75; CrI 0.31–1.78), GVHD (OR 1.21; CrI 0.59–2.47), and length of hospital stay (OR 0.90; CrI 0.29–2.72) were similar in both groups. We found weak evidence of heterogeneity amongst the studies and moderate publication bias.

**Conclusion:**

There is weak evidence in favor of primary oral vancomycin prophylaxis in preventing CDI in HSCT patients without significantly affecting secondary outcomes. Further studies are needed to provide stronger evidence, and assess long-term safety, potential effects on antimicrobial resistance and influence on gut microbiome.

**Disclosures:**

All Authors: No reported disclosures

